# Expanding DNA database effectiveness

**DOI:** 10.1016/j.fsisyn.2022.100226

**Published:** 2022-04-05

**Authors:** Ray A. Wickenheiser

**Affiliations:** New York State Police Crime Laboratory System, Albany, NY, USA

**Keywords:** DNA database, Indirect comparison, Y-STR Searching, Mitochondrial DNA searching, X Chromosome searching, Kinship analysis, Investigative genetic genealogy, Forensic genetic genealogy, Genetic genealogy, Forensic DNA, Unidentified human remains, Universal DNA Database, Enhanced DNA Indirect matching

## Abstract

DNA databases effectively develop investigative leads, with database size being directly proportional to increased chances of solving crimes as demonstrated by a business case including a universal STR database example. DNA database size can be expanded physically by increasing the number and type of qualifying offenses, adding arrestees, or moving towards a universal database. The theoretical size of a DNA database can also be increased scientifically by using the inherent nature of DNA sharing by biologically related individuals by using an indirect matching strategy including Partial Matching, Familial Searching, and Investigative Genetic Genealogy (IGG). A new strategy is introduced using areas of shared DNA as a search key to locate potential relatives for further kinship evaluation. New search key strategies include Y-STR, mtDNA, and X Chromosome searching to locate potential relatives, coupled with kinship and genetic genealogical research, as well as expanded use of unidentified human remains (UHRs).

## 1 Introduction

Strategies to solve crimes currently include developing DNA profiles from crime scenes and comparing them directly to known suspects, and to DNA databases of known individuals and other unsolved crimes. For major criminal cases, the strategy has been expanded to include indirect comparison, using such techniques as Partial Matching, Familial Searching, and investigative or forensic genetic genealogy (IGG or FGG). The term IGG will be utilized in this discussion. Regardless of the strategy, if a database is used, larger databases increase the rate of success, as will be demonstrated by a business case, as well as a universal STR database example. Making a business case for an initiative is commonly used terminology in business for providing an analysis of cost and benefit, to clarify options so the most beneficial option can be selected. Law enforcement, forensic science, and other government services have relatively fixed budgets; hence they should evaluate options for the most cost effective outcomes. Therefore, business cases are provided herein to demonstrate the value proposition provided by the option being evaluated.

A new strategy discussed herein includes expanding the size and nature of the DNA profile developed from biological materials left by perpetrators at crime scenes. These expanded DNA profiles will enable increased comparison to different perpetrators and different crime scenes to each other, and potentially to databases of known individuals. Expanded DNA profiles refers to DNA typing beyond the core CODIS (Combined DNA Index System – FBI, United States of America) autosomal loci. Expanded profiles include Y-STRs (Short Tandem Repeats from the Y Chromosome), mtDNA (mitochondrial DNA), the X chromosome, expanded autosomal DNA including SNPs (Single Nucleotide Polymorphisms) and WGS (Whole Genome Sequencing).

X-STRs are not currently routinely analyzed nor used for forensic profiling. STR profiles utilized for the core CODIS loci originate from the 22 autosomal loci, which do not include X and Y chromosomes. The X and Y sex chromosomes have different modes of inheritance; hence they offer different information that can be linked between biologically related individuals. This strategy increases the discrimination of a kinship evaluation as well as a search for related individuals. The same types of DNA polymorphisms are present in the X, Y and autosomal chromosomes. While currently WGS in the forensic context is used to extract SNPs for the purpose of upload to genealogy databases, WGS could also be used to provide STR profiles for CODIS, as well as Y and X STRs. WGS may be the ultimate forensic profiling solution for some forensic samples in the future to provide the most data from a single laboratory analytical technique, versus a scaled approach analyzing DNA in stages. If cases are determined to be committed by individuals who are biologically related to each other, this additional information may be helpful in solving previously unsolved crimes, utilizing kinship analysis, genealogical research, and family tree building.

Perpetrator DNA profiles recovered at crime scenes in criminal investigations, known as forensic profiles, represent a huge crime solving and prevention tool. As of October 2021, the U.S. National DNA Index (NDIS) contained over 14,836,490 offender^1^ profiles, 4,513,955 arrestee profiles and 1,144,255 forensic profiles [1]. As the terms NDIS and CODIS (Combined DNA Index System) are frequently used interchangeably, the term CODIS will be used for the purposes of this article to refer generally to databases of known offenders or arrested individuals, as well as forensic profiles. Those forensic profiles recovered from biological materials deposited at crime scenes have produced 587,773 hits assisting more than 574,343 investigations, which roughly equates to a 51.37% hit rate [1]. This also means approximately 556,482 forensic profiles or 48.63% are still seeking a match to provide an investigative lead to assist investigators to solve that crime. Cases that have not had a database match represent an opportunity to consider additional measures to provide an investigative lead.

The United Kingdom and New Zealand have well developed criminal investigative DNA databases. As of December 31, 2021, the UK National DNA Database had 6,829,278 subject profiles and 678,367 crime scene sample profiles [2]. While the UK hit rate has been cited to be above 60%, specific statistics are not available [2,3]. The New Zealand DNA Profile Databank possesses over 200,000 samples and over 40,000 DNA profiles from forensic samples [4]. There is a match rate of nearly 70% for all previously unsolved cases successfully linked to individuals and 30% link to another crime [4]. In 2022, the population of New Zealand is estimated at approximately 4,834,420 [5]. This indicates that roughly 4.14% of the population is contained in the database. The population of the UK was approximately 68,374,386 as of February 8, 2022 [6]. With 6,829,278 individuals in the UK National DNA Database, this equates to 10.00% of the population. The U.S. population as of February 7, 2022, was 332,485,013 [6]. With 14,836,490 offender profiles, this represents approximately 4.4% of the U.S population [7]. An increase in database size will generate a greater number of hits, however a critical factor in database effectiveness is increasing the number of profiles from the active criminal population within the database. Walsh et al. determined that while there are some apparent trends, they found no significant correlations between either the Return Index (number of hits divided by the number of samples) or hit rate and the size of the database [8].

China reportedly has over 80,000,000 profiles from boys and men in its database [9]. This wide-spread collection is motivated by the fact that males commit more crimes than females. However, database expansion was especially driven by a particularly violent crime spree in the Northern Chinese region of Inner Mongolia including the rape and homicide of 11 women and girls, including one as young as 8 years of age. In these cases, over 230,000 fingerprints and over 100,000 DNA samples were collected and analyzed. In 2016 a man was arrested on unrelated bribery charges and linked to the crimes through DNA. The suspect, Gao Chengyong, confessed to the crimes and was later executed [9]. These cases and the success of the investigation including DNA spurred state media to push for the creation of a national DNA database. Notwithstanding the success of the Chinese database in solving crime, there have also been concerns expressed regarding the potential use of DNA to identify ethnic minorities and associated human rights violations, particularly involving the largely Muslim Uyghur minority group in Northwestern China [9,10]. With DNA comes great crime solving power, but also great responsibility to protect against its potential abuse.

The preceding and following opinions and observations flow from the author's experience based on the U.S. system. Each country, state and jurisdiction must make decisions regarding the tradeoffs between public safety and individual rights, specifically regarding the use of indirect matching to provide investigative leads. Some countries may believe that only direct matching may be applied, just as they may deem various conditions are worthy of inclusion in a DNA database of known individuals. Individual American states have determined which crimes warrant submission of DNA profiles for database inclusion, and whether or not to include arrestees. They have applied the concept of proportionality to override individuals' right to autonomy to achieve greater public safety through use of a DNA database. Thirty-one American states currently include arrestees in CODIS, moving the balance towards public safety in enlarging the physical size of their database of known individuals to support criminal investigations with additional leads [11].

Expanding the footprint of DNA databases scientifically by including indirect comparisons to search for biologically related individuals is a strategy which uses the characteristic of DNA sharing among kin to identify suspects as well as unidentified human remains (UHRs). As these crimes are unsolved, not solving them risks perpetrators at large committing more crimes against new victims. It also leaves victims and families without closure and potentially living in fear, as well as families of missing individuals not knowing what happened to their loved ones. Each unsolved crime costs money and resources, as investigative costs are still incurred while crimes are unsolved. Unsolved crimes also represent a cost to the innocent, potentially facing investigative questioning and suspicion being cast upon the wrong individual and an increased risk of a wrongful conviction. Unsolved crimes diffuse investigative energy and increase costs and risks, while preventing closure.

Increasing database effectiveness by generating more investigative leads through indirect matching means that individuals who are known not to have committed the instant crime are used as an investigative tool to locate the person that did potentially commit the crime. Use of the individual's DNA profile to connect to related individuals does not incriminate the first individual, however, has perceived negative impacts by using their profiles as a means to the end of locating their perpetrating relative. Through application of the concept of proportionality, there is now a higher level of concern over infringing upon an individual's right to autonomy, by now utilizing their DNA to include related family members [12]. This greater concern on protecting rights of individuals over public safety has been incorporated into polices and laws [[13], [14], [15]]. Additional checks and balances have been added to address this increased potential threat to autonomy, including investigator education, informed consent for third party samples, confidentiality requirements, and types of crimes that will be considered [[13], [14], [15], [16], [17], [18], [19]]. Hence, policies surrounding Familial Searching and IGG reserve these techniques for major crimes rather than property crimes [[13], [14], [15], [16], [17], [18], [19], [20]]. In every technique of indirect matching, the next step is a direct comparison from the suspect sample collected from the person of interest to the crime scene sample, which serves as the ultimate confirmation or elimination of the investigative lead.

## 2 Business cases and methodology

### 2.1 Business case for expanding the size of offender DNA databases

While expanding the size of a database to generate more investigative leads makes inherent sense from a public safety standpoint, a cost benefit analysis can be utilized to demonstrate the improved crime solving capability in monetary terms. Various studies demonstrate a very positive cost saving through analyzing sexual assault kits and the use of forensic databases for direct hits [[21], [22], [23], [24], [25], [26]]. Philosophically the same cost savings can be extrapolated to include theoretical database size expansion through employing indirect matching strategies as well. A specific business case will also be provided to assess the cost benefit of indirect matching.

A large cold case project and DNA database expansion in an American state in the early years of database implementation will provide data for analysis of the impact of DNA database size. In the author's opinion, using biological relatedness to enhance DNA database effectiveness is applicable to any county, state or jurisdiction not using a universal database. While the database example is from a single forensic laboratory and state DNA database, the same expansion should produce a similar increase in positive results in other locations. Note these projects were conducted over the span of numerous years. Building DNA analytical capability is a long term investment in infrastructure, with a correspondingly significant payback.

**Fig. 1:**
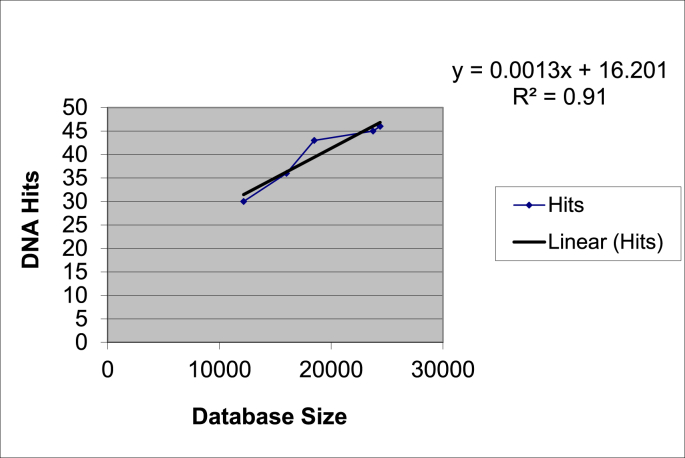
Project resolution DNA hits to Louisiana state database size 2003–2004.

**Fig. 2:**
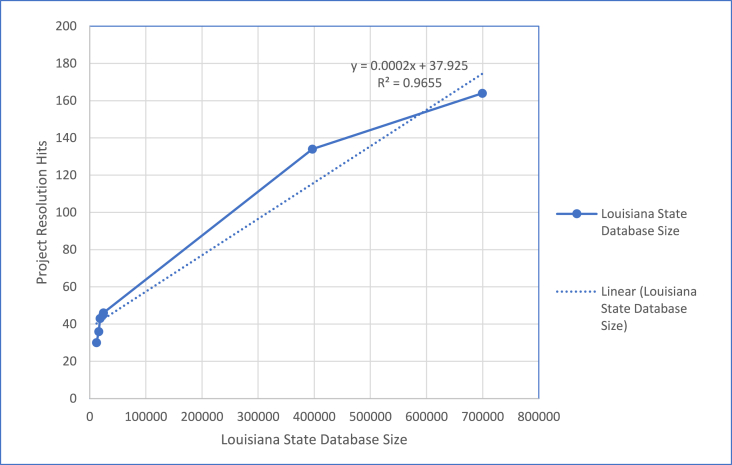
Project resolution DNA hits to database size 2003–2021.

A cold case project conducted in Louisiana termed Project Resolution generated 295 forensic DNA profiles which produced a large number of database hits over its life span [23]. Once profiles were returned from the vendor lab and entered into CODIS by Acadiana Criminalistics Laboratory (ACL), a number of DNA hits were returned immediately in 2003. Then, over time as the number of offender samples in the Louisiana CODIS State DNA Index System (SDIS) increased, additional matches were made as more of the originators of the DNA samples had their known profiles available for comparison to forensic profiles. As demonstrated in Fig. 1, Fig. 2, the larger the database size, the more hits occur in a relatively linear manner.

The final entry of forensic profiles for ACL's Project Resolution occurred on January 15, 2004. When the Louisiana State SDIS size was initially 12,000 offender samples, 30 hits occurred to forensic profiles. As the database size grew to 47,000 offender samples as demonstrated in Fig. 1, hits to forensic profiles increased to 36, 44, 45 and 46 respectively. This increase in DNA hits was relatively linear with an R^2^ value of 0.91. The calculated slope of 0.0013 means that for every 1000 new database profiles entered into the Louisiana SDIS, there would be approximately 1.3 hits, or a 0.13% chance of obtaining a new hit. This equates to one hit for every 769 new offender profiles entered into the Louisiana SDIS for Project Resolution alone. This does not include all of the other hits to forensic profiles which could be occurring outside of the ACL jurisdiction. Noting that DNA database samples cost approximately $30-$50 apiece to analyze [21,22], the cost of building the database was also quite cost effective. At $50 per offender sample and 769 offender samples per hit to a forensic profile, this demonstrates a cost of approximately $38,450 per hit for ACL cases alone.

As of April 6, 2011, in an update for Project Resolution outcomes, there were 134 CODIS matches in these cases to 119 offenders [23]. An update from ACL Director Kevin Ardoin on March 9, 2021 indicated that over the last 10 years an additional 30 matches have occurred, totaling 164 matches out of 285 profiles. This equates to a 58% hit rate to convicted offenders, arrestees, or to other cases. The CODIS hit rate increased from 47% to 58% in the last 10 years due to DNA database expansion, as no additional forensic (crime scene) profiles have been added to Project Resolution.

Most recently, information provided by Louisiana State Police Crime Laboratory Director Adam Becnel and CODIS Unit Manager Phillip Simmers on June 14, 2021 indicated Louisiana SDIS sizes of 396,676 on April 6, 2011 and 699,618 on June 14, 2021 (see Table 1).

**Table 1:** Louisiana CODIS database size to project resolution hits.

Date	Louisiana State Database Size	Project Resolution Hits
2003, month and date unknown	12,162	30
2003, month and date unknown	16,003	36
2003, month and date unknown	18,492	43
2003, month and date unknown	23,738	45
2004, January 15	24,379	46
2011, April 6	396,676 (b)	134 (a)
2021, June 14	699,618 (b)	164 (a)

(a) Source: Acadiana Criminalistics Laboratory Director Kevin Ardoin, April 6, 2011, and March 9, 2021 respectively.

(b) Source: Louisiana State Police Crime Laboratory Director Adam Becnel and CODIS Unit Manager Phillip Simmers, June 14, 2021.

Contrasting the two charts, Figs. 1 and 2, demonstrating the DNA Database Hits from 2003 to 2004 and 2003 to 2021 respectively, each shows excellent linearity, with R^2^ values above 0.90. Over time, the number of Louisiana SDIS offender samples yielding each forensic hit increased from 769 per hit to approximately 4945 per hit (134 hits upon 396,676 database samples) in 2011. Over the following 10 years, the number of Project Resolution hits grew to 164 hits upon 699,618 database samples, which equates to 1 hit per 4266 Louisiana SDIS samples in 2021. Fig. 1 visually demonstrates a very positive and consistent relationship between the number of database samples and the number of hits. While Fig. 2 demonstrates good linearity, and an improved R^2^ value compared to Fig. 1, the increased hits are not in the same proportion relative to the database sample number. With a slope of 0.0002, this corresponds to approximately 1 hit per 5000 database samples. The slope provided in the linear equation is very close to the direct calculation of 1 hit per 4266 offender samples (164 forensic hits to 699,618 offenders), supporting the R^2^ value of 0.9655 across the history of Project Resolution hits relative to offender database size.

The initial success rate of 1.3 hits per 1000 database samples from 2004 dropped to 0.2 hits per 1000 offenders in 2021, taken over the 17 years that have elapsed since the project concluded. This reduction in hit efficiency is not unexpected given these are cold cases committed between 1974 and 2004. Individuals arrested and convicted over the last 20 years later are apt to include individuals too young to have committed the crimes in the earlier range of the 1974-to-2004-time span. Thus, over time the number of hits on a static set of forensic profiles is expected to diminish as more youthful offenders are entered into the DNA database. The correlation of forensic hits to offender database size thereby shows a very positive yet diminishing rate of return. ACL is one of seven forensic laboratories in Louisiana, and Project Resolution was a single project within a continuum of forensic cases being queried across the state and nation. Therefore, if increasing database size results in significant hits in a single cold case project, its value is much, much greater across the entire span of forensic cases queried against it [24,25].

A calculation of the cost benefit of increasing the number of offenders in the Louisiana SDIS reveals a very positive benefit, regardless of the increased number of database samples per forensic sample hit. Utilizing the most recent cost of crime estimation of $435,419 per sexual assault, and a recidivist factor of 26.22 preventable crimes per hit [25], the return on investment is $53.52 per $1 spent (see Table 2). This equates to a return on investment of 5352%, which strongly supports the Louisiana investment in increasing the number of offender DNA profiles in the Louisiana SDIS as a crime solving and prevention tool.

**Table 2:** Cost benefit of Louisiana SDIS increase for project resolution hits.

Cost per sexual assault	$435,419
Number of preventable sexual assaults per hit	26.22
Number of hits	164
Size of Louisiana SDIS	699,618
Louisiana SDIS offenders per hit	4266
Cost benefit of Project Resolution	$1,872,336,534
Analysis cost per database sample	$50
Cost of Louisiana SDIS offender sample analysis	$33,980,900
Cost benefit per $1 spent	$53.52
Return on investment percentage	5352

### 2.2 Universal law enforcement DNA database business case

An alternative to indirect matching or expanding offender and arrestee database size is to include all individuals in the database of known individuals. This inclusion of every individual is termed a universal database. While this option is considered very extreme, it removes the potential for bias between racial and socioeconomic makeup of law enforcement and public genealogy databases. Theoretically, with all individuals’ known profiles included for comparison, no forensic profiles would not match a known individual, and hence would provide an investigative lead in every case where a forensic DNA profile was available. A cost benefit analysis can be used to determine whether this largest most extreme example of expanding database size is cost effective in solving more cases and hence preventing future crimes. A return on investment can be calculated by comparing the cost of a universal database to the savings from solving and preventing crime.

The universal STR database option describes a theoretical scenario where every individual of age in a society has their autosomal STR DNA profile in a database, which would be searched against forensic profiles. Not all jurisdictions may have appropriate safeguards to protect privacy as well as resources where healthcare and fighting poverty are competing factors. Therefore, implementation of database policies should include appropriate safeguards to ensure individual privacy is protected and public trust is maintained. Where public safety can be impacted by technology resource increases, the cost benefit dollar values provided here are compelling to provide justification for the investment.

The estimated cost of a database including all Americans would be approximately $16.5 Billion (331.4 Million Americans times $50 per sample for analysis) [23,26]. Examining the preventable crimes by solving unsolved sexual assaults can be used as a model to estimate the benefit side of the cost benefit equation. According to the FBI 2018 Crime in the United States report, 139,380 sexual assaults occurred in the United States in 2018 [27]. Palm Beach reported in their cold case project that 44% of sexual assault cases yielded a CODIS eligible profile [28]. In Detroit, their cold case project obtained 785 CODIS eligible profiles in 1595 cases tested, or 49.2% [29]. In Acadiana Criminalistics Laboratory, Project Resolution resulted in a 47% CODIS eligibility rate in sexual assault case analysis [23]. The average success rate among these 3 cold case projects is 46.7% in generating CODIS profiles from sexual assault cases. The greatest hit rate within the 3 labs was Detroit at 57.96%, which indicates that approximately 42% of their CODIS profiles did not receive a hit. Using that 42% as an unrealized hit potential among the 139,380 annual U.S. sexual assaults, this means an additional 58,539 sexual assault cases could be solved if a database was in place that included all individuals.^2^ Using the cost of $435,419 per sexual assault case, and 26.22 preventable sexual assaults per hit [25], the opportunity cost is $66.8 Trillion. With the cost of analyzing the entirety of the US population to create an all-inclusive CODIS database at $16.5 Billion and the annual cost savings of the expansion to gather hits on the missing 42% at $6.8 Trillion, then the return on investment is $4050.47 per $1 spent. This is a massive 405,000% return on investment, demonstrating the large cost utility of increasing database size on a larger and more universal scale.

As of October 2021, the NDIS contained over 14,836,490 offender^3^ profiles, 4,513,955 arrestee profiles, which equals 19,350,445 known profiles available for comparison [1]. There were also 1,144,255 forensic profiles in NDIS [1]. Those forensic profiles recovered from biological materials deposited at crime scenes have produced 587,773 hits [1], which equates to roughly a 51.37% hit rate ((1,144,255/587,773) x 100). A CODIS match to one of the cases is estimated to occur in approximately 40%–58% of sexual assault cases as demonstrated by three cold case projects, which substantiates this national hit rate [23]. These 587,773 hits on 19,350,445 known profiles yields approximately one hit per 32.92 offenders, demonstrating the elevated U.S. national hit rate relative to the single cold case project.

In the UK, people from a Black, Asian, ‘Mixed’ or ‘Chinese and other’ background were over-represented as defendants in the criminal justice system in 2019, according to Ministry of Justice data [30]. This was largely because people from these ethnic groups made up a disproportionate share of people arrested, and this carried through to the prosecution, conviction, and imprisonment stages [30]. In Australia, indigenous children are over-represented at each stage of the criminal justice system, being between 3 and 16 times more likely to be charged by police and 7 to 10 times more likely to appear in children's court than non-Indigenous children [31]. Indigenous children are 17 times more likely than non-Indigenous children to be under community supervision and 23 times more likely to be in detention, while Indigenous adults are 12 times more likely to be incarcerated than non-Indigenous adults [[32], [33], [34]]. In the U.S., the racial breakdown of the DNA database is 42.9% White, 23.6% Black, 23.2% Hispanic, 0.75% Asian. 0.38% Native American and 0.8% Other [35]. The population of the US is 62.04% White, 17.77% Hispanic, 13.26% Black, 5.63% Asian and 1.31% Native American [35]. These percentages demonstrate an overrepresentation of minorities relative to their population percentage [35].

Alternatively, IGG utilizes publicly available genealogy databases, which are overrepresented by Americans with European ancestry. In 2018, it was estimated that 60% of white American perpetrators with Northern European descent could be identified by IGG, with a prediction that within 2–3 years the percentage would top 90% for white individuals of European descent [35]. The ancestry of individuals present in DNA databases impact the investigative leads they provide, reflecting the choices of who populate them. Just as minorities are overrepresented in law enforcement databases, publicly available genealogy databases are populated by those with greater experience and contact with those respective programs. The background of individuals housed in a database accessed by law enforcement is a contentious topic, which supports the option for a universal database. While a universal database eliminates this sensitive issue, it raises other significant issues, including challenging the U.S. ideal of presumption of innocence.

Kuwait had plans for a universal DNA database, however they were cancelled [36]. Passed in 2015, the law was challenged in 2016 by lawyers in Kuwait. A year later in 2016, the country's Constitutional Court ruled that the law violated the constitution's guarantee of personal liberty [36]. In 2015 in the Kingdom of Bahrain, a national DNA database containing the genetic fingerprint of every citizen and resident living in Bahrain was planned however no published information could be found to confirm specifics [37]. In 2013, the Emir of Qatar ratified a new law establishing a national DNA database to track criminals and unidentified persons [38]. According to a statement released by Qatar's state news agency, the draft law would make “DNA tests and data kept in the DNA database to be authoritative in evidence unless proven otherwise.” [39]. Scoring 86.17% in the safety index and 13.83% in the crime index, Doha was ranked as the second safest city for the second year, according to Numbeo's Crime Index by City 2022 report [40]. Numbeo is the world's largest database of user-contributed data on countries and cities globally [40]. Countries with a higher level of autocratic control in the middle east have ventured toward a universal database, with public safety a desired outcome, as demonstrated by the city of Doha in Qatar. This increase in public safety may come at the expense of personal freedoms, although the point has been debated that rights are actually increased under a limited to STRs but widespread by population inclusion DNA databank [41].

Despite the large potential savings of a universal DNA database, one might consider it unlikely such a drastic move would be taken from a policy standpoint. One need not look further than the COVID-19 vaccination response to determine that mandating individuals to do something is difficult to achieve. The share of people partially vaccinated against COVID-19 as of February 12, 2022 is 99% in the United Arab Emirates, with 88% in China, 85% in Canada, 77% in the UK, and 76% in the US [42]. This compares to only 8% in the developing countries of Ethiopia and 7% in Nigeria [42]. Those countries with both resources and a greater leaning toward state authority claim greater rates of COVID-19 vaccination than those countries who afford their citizens a choice regarding their own self-determination or autonomy. The illustrated savings of a universal DNA database of CODIS profiles provide rationale as to why increasing database size has taken place in all U.S. States. Hence, many states have taken other measures to increase the database size through looking at biologically related individuals to reap the crime solving and public safety savings benefits without physical expansion of the number of known DNA samples.

### 2.3 Partial Matching

Partial matching is utilized by some forensic laboratories, including those within New York State as approved by the New York Commission on Forensic Science, to provide investigative leads when similar looking forensic profiles are found [43]. Partial matches occur where the DNA laboratory happens upon similar looking DNA profiles which do not fully match, meaning they do not originate from the same individuals [44,45]. Partial matches may occur by coincidence where unrelated individuals share many of the same alleles by chance, or the partial match may occur because the individuals are biologically related. If profiles where many alleles are shared are located by forensic laboratory staff, rather than ignoring the similarity, these profiles are investigated by calculating kinship statistics. If these statistics meet certain thresholds, then the investigative lead is released to law enforcement agencies, with the information that the indirect lead is not a match, but rather the perpetrator may be a close relative of that individual [43].

This method of indirect or Partial Matching resulted in the solving of a serial killer case in Suffolk County, New York [46,47]. John Bitrolff was identified and subsequently convicted of the homicides of two women in Suffolk County following a lead that occurred when a relative's profile closely compared to the unsolved crime profile obtained from semen found on the deceased (see the Case Studies section).

Partial Matching frequently occurs in the forensic laboratory setting by coincidence, rather than as a designed search. Some states, such as New York, have specific policy regarding when a partial match is to be pursued, including passing two distinct kinship statistical thresholds [43]. The New York State Police Crime Laboratory System currently does not actively search forensic profiles against each other for the presence of partial matches. It does pursue partial matches with kinship potential if they are happened upon in casework and during quality assurance measures. These quality assurance measures include comparing forensic DNA profiles against each other to locate and eliminate typographical errors, such as a single locus difference where the rest of the profile is identical.

If a partial match candidate passes the kinship threshold, a lead is provided to investigators, with information that the lead is not the perpetrator, but could be a biologically related individual. Investigators then build a family tree around the lead, and compare individuals to specifics of the crime, including sex, age, location, and any other particulars that may include or eliminate candidates from suspicion. Once a suitable candidate is found, a known sample is obtained for direct comparison to the forensic profile. This direct comparison serves as a fail-safe, ensuring that related individuals who are not the perpetrator are eliminated with 100% accuracy. An elimination of an individual who is also a relative can in turn have a kinship statistic calculated, which will provide further information to guide the investigation.

### 2.4 Familial Searching

The technique of Familial Searching includes actively searching a database of known individuals, evaluating profiles for potential biological relatedness to an unsolved forensic profile using a kinship algorithm [12,14,48,49]. Familial Searching was applied in 2002 by Acadiana Criminalistics Laboratory in the South Louisiana Serial Killer case, which was eventually determined to be Derrick Todd Lee via a trace DNA profile found on a surviving victim [23]. Craig Harman, of Frimley, Surrey, England, became the first man to be convicted using familial DNA techniques in 2004. He threw a brick from a motorway bridge which crashed through the front window of Michael Little's truck, triggering a fatal heart attack in the driver [50]. After the kinship calculation, closely matching profiles are then further evaluated for potential kinship. This evaluation frequently involves additional DNA analysis, including comparing Y-STR profiles. This additional DNA analysis uses Y-STRs and potential additional autosomal comparisons to save investigative work by eliminating coincidental similar looking profiles from those that may actually be biologically related. In this manner, false leads are minimized as coincidental autosomal DNA indirect matches are indicated when Y-STR profiles and additional autosomal loci are different, once potential mutations are accounted for. While issues have been raised regarding the ethics of examining relatives that are known not to be the preparator as a means to solve a crime [[51], [52], [53]], 12 U.S. states have determined the benefit outweighs risks and utilize Familial Searching for selected serious crimes.

The notable difference between Partial Matching and Familial Searching is that Partial Matching typically occurs by coincidence rather than a deliberate search. Once a similarity is seen between non-identical profiles, forensic scientists do not look the other way, but rather follow up on the similarity to see if there is in fact a potential biological relationship. In contrast, Familial Searching is a deliberate strategic search of a database of known individuals looking for potential biological relatives, when an initial search has not resulted in any direct one-to-one matches.

Once a lead is developed, similar to Partial Matching, investigators are provided with information that the lead is not the perpetrator. Rather, the lead is a very possible close relative of the perpetrator, usually a first order relative. This includes siblings, parents, and children. With this information, investigators build an immediate family tree surrounding the investigative lead and pursue a known sample from individuals who fit the particulars of the crime. Only once there is a direct match to a suspect is an individual considered for arrest and prosecution. As in the technique of Partial Matching, having a forensic profile thought to be that of the perpetrator generated from crime scene biological material in advance of comparison to a suspect, provides an objective means of including or eliminating new suspects with a high degree of confidence and objectivity. Generating a forensic profile in advance of known comparisons reduces potential bias.

The technique of Familial Searching has seen multiple advances since its inception, permitting expansion of the forensic profile types which can be searched, as well as improvements in indirect searching capabilities [[54], [55], [56], [57], [58], [59]]. Use of likelihood ratios was demonstrated as a superior method of Familial Searching over allele sharing [54]. Myers et al. described likelihood ratio use and performance for the California DNA database, including solving the Grim Sleeper serial killer case [55]. Likelihood ratios can also be utilized to improve direct matching of individuals in databases [56]. Mixed DNA profiles occur in many forensic samples. Combining mixture deconvolution through probabilistic genotyping with likelihood ratios expands the number of forensic profiles which can be directly and indirectly searched [[57], [58], [59]].

### 2.5 Case Studies

#### 2.5.1 Rita Tangredi and Colleen McNamee Homicides

John Bittrolff is an American convicted murderer and suspect in the Long Island Serial Killer Case, fatally beating two women to death [46,47]. Rita Tangredi was found dead on November 2, 1993 while Colleen McNamee was beaten, strangled to death, and left naked in the woods on January 30, 1994. John Bittrolff became a suspect in these two cases through the use of Partial Matching. His brother, Timothy Bittrolff, was included in CODIS for violating an order of protection. Tim Bittrolff's profile was subsequently indirectly matched to the forensic sample found on the bodies of Rita Tangredi and Colleen McNamee in 2013 [46,47]. Kinship calculations verified there was potential merit to a biological association between the similar non-matching profiles. After this kinship evaluation a report including the investigative lead was issued. Once investigators were informed that Timothy Bittrolff was very possibly a close relative of the killer, they determined he had a brother who fit particulars to the two crimes, including his nearby location. They obtained a DNA sample from Tim's brother, John. The DNA sample taken from John was analyzed and directly matched to forensic profiles found on both homicide victims. John Bittrolff was found guilty and convicted to two consecutive 25-year life sentences.

#### 2.5.2 Wendy Jerome Homicide

Wendy Jerome was the victim of a 1984 Homicide in Rochester, New York which was solved with the assistance of a lead provided by Familial Searching [60]. The case was the first of its kind in New York. Fourteen-year-old Wendy was last seen alive leaving her home to deliver a birthday card to a friend around 7 p.m. on Thanksgiving Day. She had an 8 p.m. curfew. When Wendy did not return at 8 p.m., her parents reporting her missing. Her body was found less than 3 hours later behind School No. 33 on Webster Avenue near her home. She had been raped, brutally beaten, and her throat was slit. Forensic analysis yielded a profile from seminal fluid located on her body. Numerous comparisons were made to known suspects, with no direct matches. Continuous searches in CODIS did not result in a match. Investigators never gave up on the case and submitted a request for Familial Searching when the technique became available and their sample qualified.

The investigative lead which led to the arrest of a suspect in this case came in 2021 as the result of a Familial Search. Within 90 days of the lead being provided, investigators conducted a study of the immediate family of the lead, obtained a DNA sample from the suspect, and found a direct match to seminal fluid found on the body of Wendy Jerome. The suspect was 20 years old at the time of the offense and lived nearby the homicide scene in Rochester. The two individuals did not know each other. Shortly after the homicide, the suspect moved away from Rochester. He was arrested in Florida, where he had been living since his departure from the Rochester area. The trial is pending in this case.

#### 2.5.3 Minerlitz Soriano Homicide

On November 29, 2021, police arrested a man on murder charges for the homicide of a 13-year-old girl, more than two decades after her body was found in a dumpster in the Bronx, New York [61]. Minerliz Soriano went missing on February 24, 1990. Her body was found 4 days later wrapped in a plastic bag in a dumpster. A forensic profile developed from semen located on her body had been entered into CODIS and had not resulted in a database hit. Investigators applied for Familial Searching to assist in solving the case. Familial Searching provided an investigative lead to a first order relative. Police built an immediate family tree using that relative, which included the suspect of the homicide. A sample was collected from the suspect, which directly matched the forensic profile from the semen on Minerliz Soriano. A trial is pending.

### 2.6 Investigative Genetic Genealogy

Investigative genetic genealogy (IGG), also known as forensic genetic genealogy (FGG), involves generation of a single nucleotide polymorphism (SNP) profile from an unsolved forensic case sample and entering it into a consumer genealogy database. Extended family trees are built using potential relatives surfaced from that search, seeking to locate a potential relative for direct comparison to the perpetrator's profile [12,62]. One of the first such cases was that of the Golden State Killer, who was identified as Joseph DeAngelo. DeAngelo committed over 13 homicides, over 50 sexual assaults and over 200 burglaries between 1974 and 1986. Notably, the case was solved with 68 days of genealogical research fortifying investigators efforts. DeAngelo had previously evaded identification for over 40 years of traditional investigation without the use of the expanded powers of DNA indirect comparison. Therefore, greater use of DNA tools including indirect matching techniques have tremendous benefits to enhance investigators' power to include and eliminate suspects by casting a wider net than direct comparison to known samples.

The difference between Familial Searching and IGG is that Familial Searching uses the autosomal core CODIS STR (short tandem repeat) loci and law enforcement offender and arrestee databases to search for related individuals. IGG utilizes a different type of DNA marker known as SNPs (single nucleotide polymorphisms) and searches for biologically related individuals in consumer genealogical databases. IGG is capable of connecting 4th or 5th cousins to the forensic profile, while the discriminating power of the 20 STR core CODIS loci generally limits associations to immediate family members [12]. As a result, IGG family trees are usually much larger, but also cast a much broader reach over more distantly related individuals, with a correspondingly larger opportunity to solve the crime compared to Familial Searching.

The direct comparison of a suspect's known sample to the forensic sample also is the end goal of IGG, as is the case for Partial Matching and Familial Searching. Investigative leads are compared to the crime specifics and suspects are included and eliminated based on location at the time of the crime, age, and other details. The direct one-to-one match again assures that related individuals who coincidentally may be suspected of the crime are eliminated with certainty. Having the forensic profile well in advance of any suspect comparison provides an objective means of using data to include or eliminate suspects, rather than rely on eyewitnesses and fading memories.

Each type of indirect matching technique, progressing from Partial Matching, to Familial Searching through IGG, involves an increasing level of potential intrusion into the rights of individuals known to be innocent of the crime. Each technique is used because there are unmatched forensic profiles and there is not a universal database in place, in that a forensic DNA profile was entered into CODIS and a hit has not occurred. In the case of major crimes, the recidivist potential where additional crimes are perpetrated upon new victims elevates motivation to solve unsolved crimes to prevent future crimes.

### 2.7 New techniques for indirect matching

New tools proposed herein include Y-STR Comparisons, mtDNA Comparison, X Chromosome Comparison and expanded use of unidentified human remains. As demonstrated in the business cases above, expanding the DNA database size to increase the chances for an investigative lead is extremely cost effective in preventing future crimes. Therefore, additional methods to expand the DNA database using indirect matching will be explored to realize more of this demonstrated potential. Along with that exploration is an ethical analysis for implications of the expanded search.

Previous methods of indirect matching, including Partial Matching, Familial Searching and IGG, include comparison of the forensic profile to databases of known individuals in a search for biologically related individuals. Y-STR Searching, mtDNA searching and expanded loci searching introduced here all share a similar process, however propose to compare expanded forensic profiles to each other to search for biologically related perpetrators. Individuals who discard biological materials at the scene of a crime they have perpetrated do not have an assumption of privacy [12]. Therefore, different types of DNA that are shared by biologically related individuals can be used as a sorting mechanism to highlight potential crimes committed by related individuals. These crimes can then be further scrutinized for potential biologically related individuals by conducting kinship analysis on autosomal loci. If biologically related individuals are located, this investigative information may be of some value where each of the cases remains unsolved. However, if any of the linked cases have a hit to a known individual or have been solved by other means, the unsolved case now has a lead that can be pursued through family tree building, much in the same manner as Partial Matching, Familial Searching and IGG.

The proposed new combination and expansion of current techniques promises to reveal new previously undetected familial relationships, which in turn will provide new investigative leads to solve unsolved cases. This proposal is to use areas of DNA that can be compared to each other for matching, such as Y-STRs, mtDNA and X Chromosome profiles. This comparison will select for related individuals as they will share the same Y-STR and X Chromosome profiles (for males) if related paternally or the same mtDNA if related maternally. These areas of shared DNA are used as a search key, to then enable a second comparison of individuals who share the same key, so the closeness of their relationship can be determined by a kinship analysis. Techniques of direct comparisons of Y-STR and mtDNA, kinship analysis and indirect matching are commonly in use in forensic cases [63,64]. Combining them in this novel combined flow is a new application of these existing methods.

#### 2.7.1 Y-STR DNA comparison as a key for indirect matching

Y-STRs can be used as a key or search tool to locate individuals who may share common ancestry for cases. In many forensic cases, particularly those where a sexual assault has occurred, a Y-STR profile may have been generated as well as an autosomal STR (aSTR) profile. Forensic Y-STR profiles are compared to each other, and those cases with the same Y-STR profile are then surfaced for further examination. In all forensic case flows, aSTR profiles are compared for a direct match to determine possible common origin or an identical twin as a source. If any cases share the same Y-STR profiles and have a different aSTR profile, there may be significant investigative information that can be provided.

Y-STRs are Short Tandem Repeat areas of DNA variability on the Y (male) chromosome. A human male individual normally has only one Y chromosome, hence there is typically only a single allele per locus or area of DNA being examined. Y-STRs are inherited paternally, that is through the father's family line. As male relatives in the same paternal line will have the same Y-STR profile until a mutation occurs to change that profile, the same Y-STR profile is a means to associate male relatives.

The next step is to utilize kinship analysis to evaluate the potential relatedness of cases with the same Y-STR profile. Kinship analysis tools currently in use include those supplied in the CODIS suite of tools and the KinCalc tool provided by Steven Myers of California Department of Justice, as well as many proprietary programs [65,66]. The kinship analysis will determine whether the common Y-STR profile occurred by chance or may be due to biological relatedness. The results of the kinship analysis will determine the next steps, depending on how closely related the cases are to each other.

The kinship analysis may indicate an immediate family member, such as a sibling or parent-child relationship. Linking unsolved cases put investigators together to share case commonalities that may lead to opportunities for suspect development. This may include potential common surname and kinship, which in turn could assist in solving previously unsolved cases [67]. If one of the cases linked has a solid suspect, as would be the case with a direct aDNA match, preparation of an immediate family study would ensue. Candidate family members would be compared to crime scene particulars, including age, location and sex of suspect.

If the kinship analysis indicates a more distant relative, genealogical researching techniques could be employed to develop leads for relatives as distant as 3rd, 4th and 5th cousins. Additional forensic biological sample is required for the generation of the SNP profiles required for IGG. As a practical consideration, many perpetrators commit crimes near the location where they live, such as the Golden State Killer [12,46,47,60,61]. Many parts of North American were settled with small groups of families and friends, and many areas have a small group of founders, including common family names. Common geography, modus operandi and combined resources may move the joint investigations forward with the new lead. Any candidates will be subsequently sampled for direct DNA profile comparison, as is practiced with the other indirect matching techniques described above, Partial Matching, Familial Searching and IGG.

A case example is used to illustrate Y-STR DNA comparison as a key for indirect matching. In this example, 2 biological full brothers Bob and Doug sharing the same mother and father have each committed a sexual assault. One brother Bob also committed a burglary, for which a known sample was collected and entered into CODIS known offender index. Forensic profiles were developed for each sexual assault case and entered into CODIS. In the subsequent search, Bob and Doug's crime scene profile was searched and Bob's forensic sample matched to the known sample Bob provided after his burglary conviction. In this scenario, Bob's case would be solved and Doug's case would remain unsolved, as there is no known sample for Doug in the database. Without a partial match or request for Familial Searching, Doug's case would remain unsolved, and Doug could continue to offend.

With a Y-STR search however, as Bob and Doug are full sib brothers, they share the same Y-STR profile, hence their two separate cases would be indirectly matched. The next step would be a kinship analysis. As approximately one half of their aDNA is in common, the resulting kinship statistic would demonstrate a likely full sibling relationship. This investigative information would be provided to investigators, who in turn would develop an immediate family tree to the known individual Bob. This family study would very likely include Bob's brother Doug, who would be compared to the particulars of the crime. Assuming that information fit, Doug would be approached for a DNA sample for direct comparison to the forensic sample. With a direct match, prosecution would begin for Doug, as a case that was given a lead through indirect Y-STR matching.

#### 2.7.2 Mitochondrial DNA comparison as a key for indirect matching

The concept of mtDNA searching is very similar to Y-STR searching described above. The principal difference is that rather than using a Y-STR profile as a key to locate possible related individuals, the mtDNA profile is utilized. As mtDNA is inherited maternally, all individuals in the same maternal family line will share the same mtDNA, until it mutates and becomes a non-matching profile. Mitochondrial DNA is used as the key or biological commonality to locate potential cases that have related perpetrators. This selection process then enables kinship tools to be then employed to evaluate the candidate cases to determine if the mtDNA profiles are closely related enough to determine who the perpetrators might be. Additional DNA testing may be conducted, including Y-STR, autosomal DNA and SNPs to enable direct comparison. As described above with Y-STR keyed searching, if the kinship comparison indicates a close family relationship, this information may be sufficient to be reported to investigators. If the kinship analysis indicates a more distant biological relationship, genealogical research may be employed, particularly if there has been a CODIS match to one of the indirectly matched cases. Each case would be evaluated on a case-by-case basis, but as with Y-STR searching, bolstered if any of the cases with the same mtDNA have resulted in a DNA match in other independent comparisons, such as STRs using CODIS or via direct comparison to suspects.

#### 2.7.3 Expanded loci, including X Chromosome, SNPs, and Whole Genome Sequencing

Besides generation of Y-STR and mtDNA profiles, analysis can be performed using the X Chromosomes, SNPs and WGS on forensic samples. The X chromosome has a different inheritance pattern in that it is inherited maternally for males, who carry only one X chromosome. For females who have two X chromosomes, they inherit one from each side of their family, receiving one X from their father and one from their mother. As males are suspects in the majority of crimes, particularly for sexual assaults, the maternal inheritance pattern provides another mechanism which can be used as a searching key for potential biological relatives.

As currently performed for IGG, SNP profiles can be entered into consumer genealogy databases and indirect matches found with biologically related individuals [12,62]. These indirect matches to known individuals provide the basis for genealogical research, which utilizes ascending family trees back in time to include the most recent common ancestor (MRCA) between the individuals and the forensic profile. The family tree is then built out to present day to include descendants of the MRCA. This enlarged family tree is examined for overlap where maternal and paternal branches coincide in a single family unit, which indicate the parents of the forensic sample provider. The immediate family of this family unit is then examined for individuals with a potential link to the crime scene, including geography, suspect age, sex and other specifics. Known samples are collected for direct comparison to the forensic sample, which then are confirmed or eliminated as potential suspects.

SNP profiles from forensic samples can also be compared to each other, providing an enhanced kinship analysis beyond that of forensic kinship tools using only STR data. As SNP comparison tools used in genealogical websites are quite advanced and SNPs provide many datapoints to search for and compare areas of DNA shared between individuals, they offer the ability to connect relatives as far afield as 4th and 5th cousins. Therefore, developing expanded DNA profiles provides assistance in kinship analysis well beyond first order relatives, making a compelling case for additional analysis for forensic samples.

WGS is emerging as having greater success with environmentally challenged samples and those from bone and hair [68]. SNP profiles can be derived from the WGS results, and in turn searched using IGG using the flow outlined above. WGS results can also provide X, Y and autosomal chromosome data, therefore there is support for gathering all of the data possible for challenged or minute samples. As forensic laboratories evaluate future analytical technology to apply to forensic samples, while the CODIS loci analysis and searching are the best initial strategy, gathering other genetic data in a single analysis could be a future means for evidence optimization when a CODIS match is not obtained.

Maternally and paternally inherited DNA are part of a shared DNA toolkit to examine biologically related profiles using various dimensions to determine potential relatedness. Immediate family members could be better targeted using a combined Y and X chromosome search or also in combination with mtDNA. More distant relatives could be selected for on either side of the forensic sample's family tree by using paternally and maternally inherited DNA searching strategically. These search keys could be used simultaneously or sequentially in various searching strategies depending on the case, sample, and available validated technology, with the end goal of suspect development and a direct comparison.

It may very well be that these markers and techniques have been used in the past for specific cases. Many forensic laboratories likely have a number of forensic samples which have both Y-STR and autosomal profiles, and in rarer cases mtDNA profiles. They very possibly have not used DNA markers in this combination of indirect matching and kinship analysis, where first a portion of a DNA profile is used as a search key, then followed up with a kinship analysis to evaluate biological relatedness. Searching for biologically related individuals has been done on a case by case basis in Familial Searching and IGG, however not as a matter of normal case flow by comparing all crime scene profiles amongst each other.

Regardless of the forensic DNA technology which emerges, maximizing the value of evidence is the forensic laboratory's mission, hence forensic samples should be subject to expanded typing and searching particularly when a CODIS hit does not occur and the crime remains unsolved. This searching would be facilitated by a database for forensic samples with expanded profiles. A tiered searching strategy would begin with CODIS STRs and flow through an array of searches to exhaust potential leads at the limit of IGG. Any matches found in one case in turn provides a known relative to connect an unsolved case which indirectly matches. Comparing forensic cases to each other is further optimized when more cases can be compared to each other in the search for biologically related individuals. As with direct comparisons, the larger the number of comparisons, the more leads can be generated, including using indirect matching. Hence, the case is made for maximum profile generation and comparison of forensic profiles for maximum crime solving and prevention.

A tabular comparison of the techniques of Partial Matching, Familial Searching, IGG and Y-STR Searching Indirect Matching is provided, to enable comparison in how the method is initiated, the DNA type, database and method of comparison used, the estimated chance of success, and its general usage (see Table 3).

**Table 3:** Comparison of partial matching, familial searching, IGG and Y-STR searching indirect matching.

Technique	Partial Matching	Familial Searching	Investigative Genetic Genealogy	Y-STR searching and other indirect matching techniques coupled with kinship analysis
**How method is initiated**	Random or semi-randomly occurring	Targeted search	Targeted search	All forensic profiles proposed
**DNA type used**	STRs	STRs, frequently followed up with Y-STR comparison to limit adventitious matches	SNPs after STRs do not directly match	Y-STR or other search key, followed by STR comparison and kinship analysis, followed by IGG depending on kinship analysis outcome
**Database used**	No database search necessarily occurs	CODIS	Consumer Genealogy Databases	Forensic profiles are be compared directly to each other enabled by a basic spreadsheet program or more advanced database tools
**Comparison**	Samples are compared one against another and evaluated with kinship analysis	A kinship algorithm calculates a likelihood ratio (LR) statistic for every profile in the database against the forensic profile and results are sorted from highest LR to the lowest	All profiles in the database are compared against the forensic profile for areas of shared DNA, measured in centimorgans (cM). Indirect matches are provided in order of highest matching cM value.	Y-STR or other search keys are compared among forensic profiles looking for matching profiles. For profiles with the same key, then the autosomal STR profile is evaluated using kinship analysis. Immediate family members are reported. More distant relatives are considered for IGG
**Estimated chances of success**	Varies widely based on similarity of profiles to each other; can be scrutinized by kinship analysis	20–25% [48]	60% or more depending on size of database and amount of genealogical research conducted [52]	20% or more initial success on all cases based on Business Case provided using identical twin extrapolation, then IGG success on remaining targeted major cases
**Usage**	Randomly occurring in casework, with some laboratories having established policy if profiles that appear to be similar are found	12 American states and numerous countries, UK, Australia; prohibited in Maryland and District of Columbia (U.S), Germany, and some EU countries [48]	Utilized in over 33 U S. States, Canada, Sweden, enabled by Maryland State legislation with judicial oversight and approval; prohibited in Germany [14]	Novel application of existing methods of comparison of profile keys, coupled with kinship analysis. Each component of the technique is separately utilized in many forensic laboratories

### 2.8 Unidentified human remains

Unidentified human remains (UHRs) have significant potential as sources of DNA to solve unsolved forensic cases. DNA mixtures are present in many forensic cases and represent issues in deducing single source profiles that can be attributed to a putative perpetrator. UHRs typically have significant amounts of DNA and are single source, which frequently makes them easier to analyze when samples have not been subject to environmental insults that cause significant degradation. Forensic value may be added to UHR cases by identifying these individuals, providing closure to families, friends and loved ones. Identifying UHRs may also help solve crimes where the UHR is a victim of a homicide, pointing to a spouse or associate with a motive.

Increased generation of DNA profiles from UHRs may also close unsolved crimes through direct and indirect matching to forensic profiles. Individuals who live high risk lifestyles are more often involved in committing crime and also have an elevated risk of falling victim to crime, as well as potentially linking their family members and associates [12,23,69]. Therefore, developing profiles from UHRs and comparing them to forensic profiles directly and indirectly adds significant additional crime solving potential.

Since 1980, 73,058 people have been buried in mass graves on Hart Island, New York [70]. An estimated 1200 of these individuals remain unidentified [71]. Hart Island is located off the cost of New York City and is the location that many UHRs and unclaimed deceased are buried for the nearby large, densely populated urban area. An estimated 1000 UHRs occur annually in the U.S [72]. The DNA of these UHRs and the many like them in other jurisdictions potentially hold a key to closure of crimes and identification of bodies. Whether the originator of the UHR is identified or not, if they are matched to an unsolved crime, this will provide closure and critical information to that forensic case. As investigative leads, UHRs can be turned into known individuals through investigation, who in turn can be linked to other unsolved crimes through indirect matching.

Expanded DNA profiling is proposed for consideration for both UHRs and forensic samples. UHRs provide a window into the known side of the matching equation, opposite the forensic profiles they are matched against, enabling expanded comparison potential. UHR profiles may directly match forensic profiles. UHR profiles may also be indirectly matched to forensic profiles, or against other known individuals, creating more opportunities to identify the UHR, and to solve unsolved crimes. They may also provide the missing link to another perpetrator through an indirect match between both a forensic case and a known individual. In numbers there is great crime solving potential.

UHR identification has few detractors. UHRs are currently used to compare against unsolved crimes. Expanding this use is a potential increased infringement into the rights of an individual, however they are deceased. As such, by applying proportionality, the rights of current and future crime victims are weighed favorably towards using UHRs to solve crime through increased DNA profiling.

### 2.9 Business case for expanded DNA indirect matching (EDIM) using identical twin extrapolation

A key question regarding the potential success of searching for family members is the number and type of biologically related individuals found in a database population. This is a particularly challenging question to answer if one seeks to determine an estimate by looking directly at specific individuals and family units. Database information is subject to privacy issues and as such is not readily accessible. As a result, providing an estimate using other information is preferable. Therefore, a proposed mechanism to estimate the number of siblings is provided utilizing the observed number of identical twins.

Any time there is a potential duplicate in the CODIS database, particularly when that sample originates from an individual with a different name, there is a quality control check to ensure there has not been a sample switch, or other quality issue. Identical twins are a well-established occurrence in humankind, with known incidence levels. Therefore, using this known incidence of identical twins, the number of identical twins identified in a database can be used to extrapolate the potential total number of siblings. This in turn provides an estimate of the likelihood of solving a case using indirect matching. This estimate is a very conservative estimate of case solving potential, as it does not include the additional mechanism of indirect matching of parent-child and other familial relationships.

In 2017, there were 536 identical twin pairs and 6 sets of triplets in the NY State DNA database, reported at the NY Commission on Forensic Science DNA Subcommittee meeting [73]. All of these individuals had committed qualifying offenses for entry into the NY State DNA database. At that time, the NY State DNA Database had approximately 700,000 individuals in the searchable Offender Index of CODIS. This index includes convicted offenders, subjects and multi-allelic offenders. The incidence of identical twins is relatively stable, at 3 to 4 sets of identical twins per 1000 live births, which is approximately 1 in 250 [74]. The rate of identical triplets is said to range from 1 in 60,000 to approximately 1 in two million [75]. If 536 identical twin pairs were present in 700,000 thousand database entries, then one would expect to see approximately 134,000 sibling pairs in the database (536 × 250). While the incidence of identical triplets varies widely, it does provide general support for a high level of siblings present in the database. The estimated rate of sibship is 1 in 5.48 individuals (700,000/134,000) in the NY State Database. This incidence of sibling pairs would provide a likelihood of finding an indirect match of a sibling at 19.14% ((134,000/700,000) X 100). This very rough sibship rate estimate provides support for the concept that biologically related individuals occur frequently in forensic DNA databases, through use of the occurrence of identical twin pairs to estimate the expected number of sibling pairs.

Three extensive cold case projects provide an average 51.18% hit rate on CODIS entries [23]. If this hit rate can be increased by a further 19.14% to 60.00%, then a very large cost savings can be realized by implementing indirect matching using Y-STR searching for siblings alone. Savings will be even greater including parent-child relationships, as well as more distant biologically related individuals utilizing IGG.

The estimated number of sexual assault hits per year nationally is 49,964 [23]. If this number was increased by 19.14%, that means an additional 9563 sexual assaults could be solved nationally each year using Y-STR searching. The cost of a single sexual assault is estimated to be $435,419 [25]. Solving each sexual assault has been estimated to prevent an additional 26.22 sexual assaults [25]. The savings from solving 9563 sexual assaults is $109.18 Billion (9563 sexual assaults solved annually X $435,419 cost per sexual assault X 26.22 prevented sexual assaults per solved sexual assault). Using this same rationale, the solving of 9563 sexual assaults would serve to prevent an additional 250,741 sexual assaults (9563 additional solved sexual assaults X 26.22 prevented sexual assaults per solved sexual assault), by apprehending perpetrators earlier in their offending trajectory. This significant cost saving and crime prevention demonstrates the utility of applying indirect matching to solve unsolved cases.

## 3 Results

**Fig. 3:**
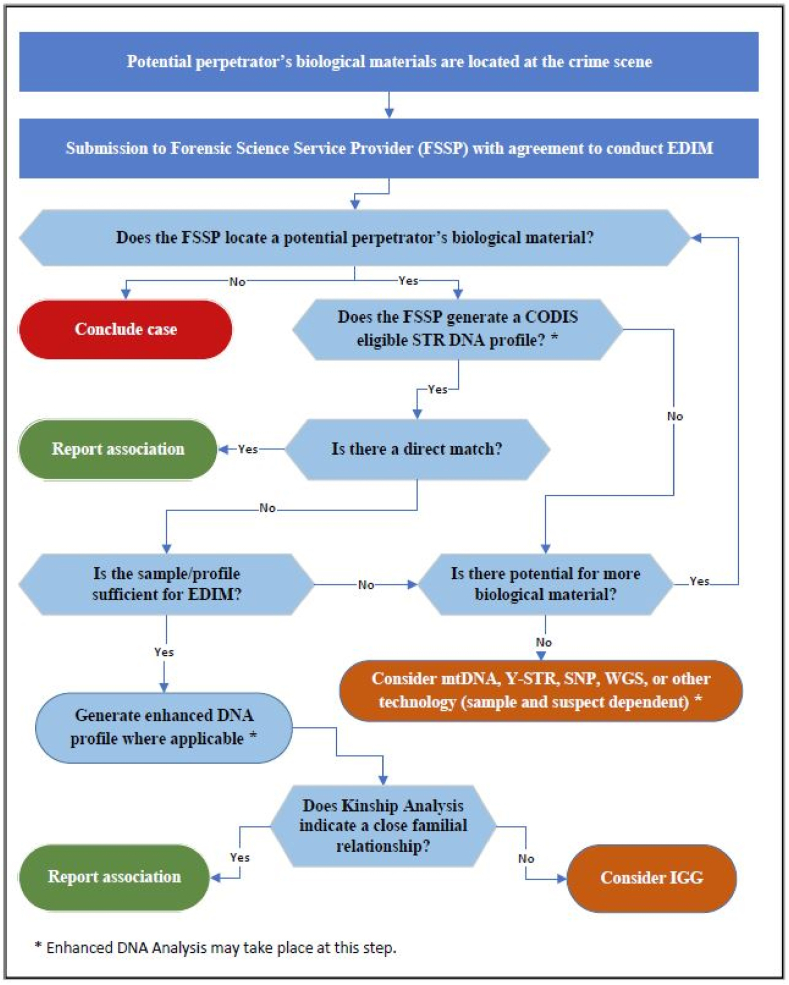
Proposed Expanded DNA Indirect Matching (EDIM) workflow.

A workflow is proposed to increase the number of investigative leads through expanded forensic DNA profiles and indirect matching (see Fig. 3).

Development and consistent use of common terminology promotes improved communications in the growing field of indirect matching and associations. Therefore, suggested common terms and definitions are provided (see Table 4).

**Table 4:** Terminology for indirect matches (associations).

Candidate or investigative lead: an indirect association that has been evaluated and has passed predetermined statistical or other criterion that could indicate that two or more cases were committed by biologically related individuals. This individual did not commit the crime but may be biologically related to the individual depositing the forensic sample in question.
Direct match, hit or association: the DNA from one profile entirely matches that of another DNA profile, with no differences. DNA profiles that are the same originated from the same source, or an identical twin with a very high level of confidence.
Expanded DNA Indirect Matching or Indirect Associations: use of an expanded DNA profile beyond the aDNA CODIS core loci, including but not limited to Y-STR profiles, mtDNA profiles, X Chromosome, and SNP profiles as used in IGG; coupled with kinship analysis, to develop investigative leads through identification of biologically related individuals.
Indirect match, hit, or association: the use of the characteristic of DNA that biologically related individuals share DNA in known patterns according to the level of their relatedness. These known patterns of transmission can be used to locate relatives through similarities shared between their profiles via a database search or direct comparison.
Search key: a component of a DNA profile that is shared among relatives which can be used to locate and evaluate cases and suspects which do not directly match to determine if they potentially involve biologically related individuals. Search keys include but are not limited to Y-STR profiles, mtDNA profiles, X Chromosome and SNP profiles as used in IGG.

## 4 Discussion

The mission of the forensic laboratory is to maximize the value of evidence, which is performed through developing DNA profiles which can be used for associations to known individuals [76]. Use of CODIS has been a game changer in terms of increasing the effectiveness of investigations through the use of a database of known individuals to solve cases by providing forensic associations [1]. Forensic science service providers have an obligation to provide best evidence, which includes making the most of the evidentiary material found at crimes scenes in terms of specificity, likelihood in providing a positive comparison, and timeliness [23,76]. While traditionally many forensic DNA laboratories have not proceeded beyond utilizing the CODIS STR profiles for direct comparison of forensic profiles to known samples, techniques using indirect matching have effectively expanded the DNA database scientifically by utilizing shared DNA as a search tool. Use of related individuals provide additional leads that can be used by investigators to triangulate to the individual who deposited the DNA, which is confirmed by a direct comparison between their known DNA profile and the forensic profile. Use of indirect matching increases the likelihood of providing an association, and therefore increases the value of forensic evidence.

From a bioethical standpoint, increasing the size of DNA databases includes some ethical and policy decisions. Hence the concept of proportionality has been utilized to weigh competing issues, where there is an ethical and economic trade-off between benefits and risks [77]. The doctrine of double effect is an example of proportionality, where if doing something ethically good has a resultant bad side effect, the original act is deemed ethically acceptable provided the bad side effect was not intended, even if it was foreseen [12,78]. Obtaining an oral or buccal swab for DNA analysis represents a physical intrusion, albeit a fairly minor one as there is limited negative impact on the individual. Developing known DNA profiles for comparison to forensic profiles infringes on the rights of an individual to not self-incriminate themselves and has the potential to reveal sensitive genetic information. Autonomy is a fundamental bioethical right, where individuals have the right to make the choice with their own body and information derived from it, which includes whether they should undergo DNA analysis or not [77]. These negatives are weighed against the positives, including crime solving and preventing potential of comparing forensic profiles against known offenders and arrestees.

The direct expansion of DNA databases by adding offender profiles has been demonstrated by a very positive return on investment using a single cold case project in Louisiana. Hit rates for forensic samples range from 36% to 57.96% for three American cold case projects, which include the Project Resolution project from Louisiana, and projects in Detroit, Michigan, and Palm Beach County, Florida [23,28,29]. This begs the question as to what can be done to assist in solving the remaining 42–64% of cases that are not hit upon. Thirty-one of 50 states have expanded their state SDIS to include arrestee database samples [79]. The number and type of qualifying offenses can be further expanded to increase database size. Samples which are legally owed by offenders but not yet collected and entered into databases should be followed up on to increase database size by applying existing laws more effectively [80]. Crime perpetrators who are aware that DNA could implicate them are motivated to avoid DNA sampling, thereby increasing urgency to follow up on uncollected DNA samples. Expanding databases scientifically through indirect matching has the potential to increase the theoretical database size exponentially, however comes at a cost of new processes and ethical implications.

The business case for indirect matching provided above demonstrates the potential to save over $4000 per $1 spent using only the crime of sexual assaults as a model, omitting homicides and other crimes which could be solved in the savings estimate. The preventative aspect of recidivist crime is also a large opportunity for crime solving and prevention, as well as increasing rehabilitative potential by interrupting criminals earlier in their career where such efforts can have greater impact. This public safety, cost of crime saving, preventative, and rehabilitative aspect of larger database use competes against encroaching on individuals’ rights to privacy. While safeguards to privacy through confidentiality protection can mitigate some concerns, current CODIS requirements include threshold for entry that include conviction and arrest. Publicly available genealogy databases used for IGG include informed consent and permit law enforcement access equal to that of the general public. The balance of these measures leans in favor of responsible expanded use of forensic samples within the existing framework of CODIS and IGG.

One of the features of law enforcement known DNA databases which is viewed negatively is the racial and socioeconomic makeup of convicted and arrested individuals that populate them. It is very noteworthy that victims of crime live in the very neighborhoods and demographic of those committing the crimes and more likely in the same racial and socioeconomic group [12,69]. Therefore, not including individuals as potential suspects based on the background of the suspect disproportionally negatively impacts victims of the same demographic. As DNA is deposited at the crime scene and analyzed prior to development of a suspect, it is an excellent means of objectively attempting to solve that crime, thereby limiting potential bias. Using proportionality as a yardstick, the negative of one group being disproportionally represented in a database may be weighed against the potential to solve more cases impacting that same group's victims. It is very important that all sexual assault cases are analyzed forensically soon after the crime occurs, which is dictated by sexual assault kit submission and analysis legislation, such as that enacted in New York [81]. Inclusion of all cases and a prompt analytical response serves the entire community. Both survivor, suspect, and future victims' rights should therefore be considered and weighed using proportionality to determine the optimal balance of DNA database policy to solve crime and provide early intervention and rehabilitation to limit re-offense.

Using CODIS as the primary and central piece of the forensic DNA process flow is well established, accepted, cost effective and well supported by associative results aiding investigations [1]. Entry of a STR forensic profile into CODIS ensures that appropriate quality assurance measures and documentation are in place, as only accredited forensic laboratories have access to CODIS. CODIS is very cost effective, as has been demonstrated by numerous studies from a cost benefit standpoint [[21], [22], [23], [24], [25]]. If a CODIS match has not provided an investigative lead and a case remains unsolved, there are a number of strategies to increase the crime solving potential of forensic DNA. These include expanding the DNA database through including arrestees and a larger number of qualifying offenses. Collecting DNA databank samples owed and not yet collected from offenders and decreasing analysis time ensures that laws and current policies are providing the most benefit. Including deceased individuals in DNA databases is a mechanism to close additional cases through matches without compromising a living person's privacy. UHRs and samples from deceased individuals may also serve as a mechanism to collect databases samples that are owed.

A critical ethical question regarding the use of UHR profiles involves privacy. Currently, DNA profiles from UHRs are compared to all forensic cases. Profiles of family members are held in a separate DNA database index, which is not subject to comparison against forensic samples. Families require confidentiality, therefore their information is segregated from the UHR's identity, and is held in confidence, as their privacy is a priority. This is similar to how the survivor of crime has their name held in confidence to protect their privacy, to support them and also to encourage other survivors to come forward. If deceased, the name of the victim is typically released. This confidentiality for survivors and family members does not prevent the investigation of the case from moving forward, nor important crime solving searches from taking place.

Expanding a DNA database to include individuals who are not thought to have committed a crime via a universal database includes a significant ethical question. Should there be a barrier for CODIS entry and search to include commission or suspicion of committing previous crimes? Currently, individuals in the known sample side of CODIS have either been convicted or arrested for a qualifying offense, or have been entered as deceased individuals. With deceased individuals currently being searched, it has been determined that their privacy is not a barrier to CODIS searching, presumably because they are deceased and hence solving crimes takes precedence. What is remaining, is the question of expanding the database beyond this current status to include more individuals, which if taken to the extreme of a universal database would eliminate the requirement for expanded DNA profiling and indirect matching. The universal database concept elevates the inclusion of individuals who are not known to have committed or been arrested of a crime versus searching of individuals known not to have committed the instant crime to the same realm. If both infringements are determined to be equal, then the universal database is far more cost effective and would conceivably solve all crimes where a forensic profile is present.

It can be debated that there is potentially less detrimental privacy compromise in comparing forensic samples to each other indirectly than to the expanded universal database known samples for individuals known to not have been involved in a crime. With an indirect match of forensic cases, there now is a justifying basis to further infringe on individuals' rights to privacy by pursuing these cases for kinship and genetic genealogy analysis, potentially examining other individuals’ profiles to develop family trees. This justification supports a forensic case comparison strategy from a proportionality perspective. The doctrine of double effect is also in effect, as solving unsolved cases that have now been indirectly matched to cases that have had a direct CODIS match will have some potential negative impact on the known database individuals who will have their family subjected to additional indirect searching scrutiny. However, those known individuals will already have first committed or been arrested of an offense to be included in CODIS and second, they have been indirectly matched to a forensic case. This situation therefore provides some just cause for the intrusion, rather than a blanket comparison to universal database expansion individuals who have not committed nor been arrested for a qualifying crime.

Proportionality can be quantified by the business case to show the very high cost of recidivist crime, utilizing the no-suspect sexual assault as a model. The savings demonstrated above are substantial, well justifying the investment in CODIS as well as consideration of EDIM. Note that many other case types including homicide have also been solved using CODIS, and the commission of property crimes leads to committing more major crimes and have increased rehabilitation rates [82]. Former Saskatchewan Social Services Supervisor Rick Beretti claimed that their department could provide meaningful rehabilitation for youth in 80% of cases if intervention was early in a youth's criminal involvement [82]. Therefore, solving minor crime using these techniques should not be discounted. Minor crimes represent rehabilitation and intervention potential, rather than strict focus on an increased punitive strategy. Minor crimes represent identifying and catching a problem early, when it can more easily be corrected, like fixing a leaky roof before major structural damage has set in.

In 1999 Iceland established a near universal health care database, that was supported by 88% of its people, with only 9000 of 270,000 individuals opting out [83]. Healthcare and law enforcement are two very different types of DNA databases for very different uses, however there are unique parallels that can be drawn. A healthcare database provides information despite its risks to privacy. Knowing that a person has a predisposition to a disease could be damaging private information, private information that could be used to save the patient's life through early intervention. This has a striking parallel to information provided through forensic laboratory results, which can be used for early identification of at-risk youths for early correction through mentorship, pre-trial diversion, and correction of broken family situations. Forensic information can be used constructively versus destructively, for correction versus punishment.

Individuals living in high crime areas may more frequently experience oppression by crime versus oppression by government overreach. According to Maslow's hierarchy of needs, one's need for safety is as foundational as the need for food [84]. Crime represents a form of individualized grassroots oppression, versus broader societal oppression from authoritarianism, top down oppression, or state sponsored oppression. Serial offenders transcend race and societies; they do not discriminate. Every culture has them. The ethical foundational principle of proportionality is a guide to maximize the benefit of informed choices, while limiting the negatives through recognizing and addressing concerns.

The benefit for a developing society is that they can observe what works and what does not in so-called developed societies and choose for their people; or better yet, have the people choose for themselves what kind of society they will build for themselves and their children. Data from DNA is like fire, which can be constructively used to heat homes and cook food, but if not controlled for good can burn down one's homes, fields and forests. Whether a government is deemed too centralized, too pro law enforcement, or insufficiently so by others; if it truly supports the will of their people and the people support it, who is to say what choice is right for those people besides the people themselves? Self-determination promotes self-respect and public trust, which are the hallmarks of civilized society. These are choices that should be laid out, recognized, educated upon, discussed and debated, so the pros may be weighed and maximized, such that the cons may be recognized and minimized, empowering people to take maximum good with controlled risk to warm their hearth. Individuals in society should provide informed consent via discussions regarding the use of indirect matching, much as Icelanders did for their universal healthcare DNA database.

China is a model to demonstrate the motivation and choices to build a large DNA database that came out of the investigation of a major serial rapist and killer in Northern China, who murdered 11 young women and children [9]. The investigation included over 230,000 fingerprints and over 100,000 DNA profiles, demonstrating how a single heinous serial killer investigation and the cost of crime justifies the cost of a DNA database [9]. Metaphorically, China can burn down the human rights house of the 12 million Uyghurs, mostly Muslim, living in the Xinjiang Uyghur Autonomous Region of Northern China, or choose to warm their hearths with increased public safety [85].

In Canada following a major serial rapist and killer case, Judge Archie Campbell's inquiry on the Paul Bernardo Karla Homolka Blue Ribbon Task Force homicides of Kristen French and Leslie Mahaffy provided a number of recommendations [86]. Two of those were establishing a 30-day turn-around time for forensic analysis and creating specific dedicated Major Crimes Units to investigate major crimes. Keeping abreast of complex technology requires a significant investment, which includes ongoing education. Given the sensitive nature of investigative information, there is a critical need to recognize that family members are not suspects and their privacy must be protected. Therefore, potential family members must be engaged to assist the investigation out of trust and informed consent. All of these complex requirements for the investigation of EDIM cases dictate specialized trained units, rather than continuously educating an ongoing turnover of investigators. The later approach risks potential investigative overreach, insensitive handling, threat to privacy, and breach of public trust. The specialized information provided by Familial Searching, IGG and democratized indirect comparison through the use of search keys and kinship analysis, requires the most careful handling and educated investigator. This is a specialized skill set and perhaps personality type befitting a major crime specialty.

Human nature is to tend to wait for the levee burst as occurred in Louisiana post hurricane Katrina in 2005, rather than to build appropriately engineered water containment structures in advance of the inevitable flood. Louisiana exists in a hurricane zone. We all live in our own zone of crime or safety, an uninformed or informed consent zone of human design. As our respective societies, we should determine our own and our families’ destinies. DNA database use and EDIM should be our own design, not left to chance or chosen out of misinformation or fear of what ifs, but rather guided by deliberative debate and active, informed choices.

Recommendations for EDIM consideration include the following:
1. Expand DNA databases as large as possible using current legislation. As demonstrated by the business case, DNA databases are an excellent return on investment. This return can be increased linearly by increasing database size.
2. Consider expanded legislation to increase DNA database size, from arrestees and expanded qualifying offenses to a more universal DNA database. Consideration should be given to consider a universal DNA database that is done for simple STRs, which will then directly match to forensic samples without revealing kinship information and involving innocent kin, as occurs with indirect matching techniques. A universal STR database for known individuals is more cost effective from a profile generation and process flow perspective, treats individuals equally, solves all outstanding cases with forensic profiles, and does not discriminate based on who is in which database. From a utilitarian standpoint, a universal DNA database is by far the most cost effective, as well as balancing and protecting rights.
3. Analyze forensic samples with expanded DNA profiles. Individuals who deposit forensic samples have no right to privacy, therefore full DNA profiles should be analyzed to maximize their evidentiary potential to solve these cases, including identifying the individual if an UHR, or identifying the perpetrator if a crime has been committed by them.
4. Add UHRs to CODIS with expanded DNA profiles. UHRs are currently underutilized to solve cases and identify previously unidentified individuals.
5. Apply EDIM to expand the effective database size scientifically. Use Y-STRs, X-STRs, mtDNA, SNPs and/or WGS to compare all forensic profiles to each other. As forensic profiles can be compared to each other ethically. If any case that is linked to another that has a DNA hit or an identified individual, these can assist in lead generation for the unsolved cases.
6. The forensic profiles for unsolved cases can subsequently be compared to any individuals who have given informed consent for genealogical research to provide investigative leads to solve these cases.
7. Establish a permanent specific dedicated group of investigators, justice system officials and forensic science service providers knowledgeable in EDIM to ensure policy and procedures are maintained and followed, and privacy is protected.

The proposed indirect search technique EDIM is a novel application of existing methods, many of which are currently conducted in forensic laboratories, or applied by law enforcement using vendor laboratories. Forensic laboratories currently compare aSTR, Y-STR and mtDNA cases upon request. They also conduct searches using CODIS profiles automatically and often, seeking to link cases by direct comparisons. Kinship analysis is also conducted upon request, such as in cases of suspected forensic paternity, including cases of incest. What is new in this proposal is the indirect search performed by the forensic laboratory without a specific case request by investigators. Ongoing searching of CODIS profiles is assumed to be conducted by law enforcement investigators, as it is performed for direct comparisons. Significant additional work is required to request additional comparisons for indirect matching cases like Familial Searching, and numerous cases with solvable evidence potential may be missed where a request for this analysis has not been made. Conducting indirect comparisons between forensic cases is a new initiative forensic laboratories can conduct to increase the utility of their service offerings, without introducing new technology. It is gained by a new assembly of existing techniques of using search keys and kinship analysis. Additional DNA profile expansion is also not new, as it is conducted routinely on specific unsolved cases without concern, as biological material has been discarded at the crime scene by the perpetrator. As such, there is no right to privacy on the part of the perpetrator.

We cannot put the genetic genealogy genie back into the bottle. Genetic genealogy is already with us. Many members of the public have already had their DNA analyzed and companies are using that DNA for research. Amateur and professional genealogists are using publicly available data to construct family trees and discover relationships that have been previously unknown, or lost over time. They are also uncovering secrets, some of which individuals and society have sought to remain unknown, including unattributed parentage, such as in the case of adopted individuals. Few appear to question the rights of an individual to use publicly available data to uncover information about their own past. They do however have serious reservations regarding permitting law enforcement to use this same information and techniques to investigate crime [41,52,87]. This increased scrutiny is appropriate to ensure information is being used properly, however the situation calls for education regarding what can and cannot be done, along with corresponding policy decisions to address concerns appropriately while enabling crime solving improvements. Law enforcement conducting investigative or forensic genetic genealogy are using the exact same techniques that members of the public utilize to find a biological parent of an adopted child. Those same tools should be available to law enforcement to solve serious crimes. Victims of crime should have the same right to have their case solved as an adoptee to locate their birth parent. Future victims of crime should have a right to future safety that their crime not be perpetrated upon them where serial offenders could be stopped earlier. An excellent case example is that of the Golden State Killer Joseph D'Angelo, where early use of indirect matched could save many lives and victimization of serious crimes [12]. Incorrect suspects must be eliminated immediately, not after years of misplaced suspicion or incarceration.

Law enforcement and forensic laboratories are here for the long term. While they have an interest in each case individually, their responsibility is for their cases as a whole, and their continuity. They have accountability for the public trust. They are responsible to keep confidentiality. They are accountable entirely to the taxpayers, rather than shareholders or other potentially diverse interests. Each case investigation conducted will have its day in court if an individual perpetrator is to be arrested and charged, ensuring the system must be subject to and withstand scrutiny on a case-by-case basis. As a result, the public should have large interest that law enforcement and forensic science service providers operating under accredited systems are those conducting or overseeing EDIM and IGG. Law enforcement and forensic science service providers should be permitted to use the same technology to solve crimes as the general public and genealogical researchers utilize to find missing relatives.

Several fail-safes are built into the EDIM process. Leads generated by indirect comparison of DNA profiles are investigative information where the forensic profile has been generated well in advance of comparison to a suspect. Any suspect is compared directly for a full matching profile. Elimination of an incorrect suspect is conducted with 100% certainty. A full match comparison and inclusion of a complete DNA profile is also performed with a very high level of confidence. The fact that profiles are analyzed in advance and separately from comparisons to suspects provides neutrality and objectivity and ensures the correct perpetrator is identified. The accredited forensic system ensures appropriate audited quality systems and documentation are in place to transparently demonstrate processes, data and interpretations. This direct comparison under an accredited system by neutral scientists that can be checked by others and scrutinized in court provides a rigorous set of checks and balances with oversight by those with appropriate expertise.

## 5 Conclusion

CODIS has demonstrated its effectiveness, which should be built upon to solve even more crimes, as approximately half of forensic profiles are not matched to known individuals. A cost benefit case has been made for a larger or more universal database and for Expanded DNA Indirect Matching. The larger the DNA database, the more crimes solved, the more future crimes prevented, the more money saved. Acknowledging the issues with a universal database, the business case supports mechanisms to enlarge the effectiveness of searching to include related individuals.

Indirect matching, including novel methods of using areas of DNA shared by biologically related individuals as search keys, is a mechanism of expanding databases effectiveness without increasing the number of known samples they contain. The STRs utilized in CODIS and the SNPs employed in genealogical databases are routinely used as search mechanisms. While CODIS focusses on direct matching, it is also being used indirectly through Familial Searching. IGG has been revolutionary in employing another more informative type of DNA through SNPs to enable indirect matching between forensic samples and biologically related individuals. Other areas of shared DNA besides SNPs can be applied to forensic samples to add even more indirect matching potential. These include Y chromosomal markers which are shared paternally and mitochondrial and X chromosome DNA, which are inherited maternally,^4^ adding new mechanisms for searching. A myriad of other genetic markers can also be considered through WGS, as this expansive DNA profiling technique is not limited to a particular DNA aspect, region or type of DNA or profile.

Each of the techniques of Y-STR and mtDNA profiling and comparison, and kinship analysis, are currently utilized in forensic laboratories. Therefore, there are no new legal nor ethical issues when these techniques are applied, albeit in a new process flow for EDIM as proposed here. Genealogical databases enable searching based on X and Y chromosome information [51,52]. Education will be required to best utilize this indirect matching technique as well as dispel misconceptions, as has been the case with IGG [88].

Using forensic profiles to compare to each other has no ethical nor legal issues, as perpetrators who discard DNA at crime scenes have no presumed right to privacy. Forensic laboratories have a mission to maximize the value of evidence and law enforcement has the duty to solve crime using all legal and ethical means at their disposal, hence expanded DNA profiling on unsolved cases is well supported. Solving unsolved crimes prevents future victims, as suspects are still at large, potentially committing additional crimes prior to their identification. Business cases demonstrate a savings of over $50 per $1 spent on expanding DNA database size using a single Louisiana cold case project and over $4000 per $1 spent for a universal DNA database. A Business Case for EDIM demonstrates an estimated increase of 19.14% cases solved, which would solve an additional 9563 sexual assaults in the U.S. annually. The resulting estimated cost of crime savings is $109.18 Billion, with the potential to prevent an additional 250,741 sexual assaults. Utilizing DNA databases in a responsible manner follows guidelines currently in place for Familial Searching and IGG [[12], [13], [14], [15], [16], [17], [18], [19], [20]]. The crime solving and prevention case for responsible implementation of EDIM is compelling.

## Disclaimer

Statements and opinions expressed herein are solely those of the author and not representative of the New York State Police, the American Society of Crime Laboratory Directors, the FBI Scientific Working Group on DNA Analysis Methods, the Forensic Science Standards Board for the Organization of Scientific Area Committees for Forensic Science, or any other organization or individual the author is affiliated with.

## Dedication

This article is dedicated to Ben Wickenheiser, who passed from this world to the next on February 11, 2022, in Saskatoon, Saskatchewan, Canada. He through his commitment to ongoing education, hard work, family and community ideals has inspired and in doing so achieved immortality.

## Declaration of competing interest

The authors declare that they have no known competing financial interests or personal relationships that could have appeared to influence the work reported in this paper.

## Footnotes

1 Offender profiles include Convicted Offender, Detainee, and Legal profiles at NDIS.

2 There are many assumptions in this rough estimate, including that all cases are current, no non-Americans committed any of the sexual assaults, etc., however note this estimate is only for illustrative purposes to demonstrate the large cost benefit of larger DNA databases.

3 Offender profiles include Convicted Offender, Detainee, and Legal profiles at NDIS.

4 Note that the X Chromosome is inherited maternally for male individuals, as they have a single X chromosome. Female individuals inherit a X Chromosome from both their mother and father; hence maternal inheritance refers only to males in this instance.
